# Insomnia and Its Therapeutics

**Published:** 1891-12

**Authors:** 


INSOMNIA, AND ITS THERAPEUTICS. By A. W. MACFARLANE, M. D., Fellow of the Royal College of Physicians, Edinburgh; Fellow of the Royal Medical and Chirurgical Society of London; Examiner of Medical Jurisprudence in the University of Glasgow; Hon. Consulting Physician (late Physician) Kilmarnock Infirmary; formerly Examiner in Medicine and Clinical Medicine in the University of Glasgow, etc., etc. Reprinted from Wood's Medical and Surgical Monographs. 294 pages, octavo, cloth. New York: William Wood & Co. 1891.

There are few ills of humanity which he may be called upon to relieve, that give more trouble and anxiety to the physician than the class of symptoms known under the general head of insomnia. As it is not a disease *per se*, whose pathogenesis may be reached by direct means, the cause must be found among the numerous ailments which more or less remotely affect the nervous organism. The first chapter is devoted to a careful examination of the physiology of sleep. Attention is called to the latest studies which prove the anemical state of the brain during sleep, and a clear idea is given of its gradual effect upon its various centers. "Briefly summarized (Dr. Macfarlane says), during sleep the brain, ganglia, medulla, and cord, are in a state of repose; and the work of the econ omy is conducted with the smallest possible expenditure of energy." The gradual onset of sleep is carefully noted and intelligently stated, while dreams, their value as a means of diagnosis, somnambulism and hypnotism, are briefly dwelt upon. Much is also said about the conditions which influence sleep and sleeplessness, and interest ing cases in the author's own practice are given in a tabular form, with general directions as to treatment and removal of exciting causes.

In regard to the treatment of insomnia and its therapeutics, several chapters are devoted to the various exciting causes. Everything which bears upon the subject seems to be scientifically and fairly treated. The difference between individual idiosyncracies is abundantly recognized, and though much must necessarily be simply theoretical, the author is careful to separate the *known* from the unknown, or even problematical. Among the causes for insomnia are given: overwork, neurasthenia, spasmodic and paroxysmal neuroses, with many other affections of the nervous system, and toxic agents. Several chapters are devoted to affections of the alimentary canal; others pay attention to the circulatory system, the respiratory system, febrile diseases, affections of the urinary system, and the insomnia peculiar to females. In each of these, therapeutic formulas adapted to the case are given, where simple hypnotics may be valueless or contra-indicated.

While we discover nothing in the book which is not already known to the well-educated physician, we think Dr. Macfarlane has rendered the profession a signal service in his analytical method of grouping together the essential facts bearing upon the subject, and to the practitioner who is debarred from access to the specialist, his suggestions, directions, and prescriptions, ought to be of very great value. The author's style is that of a man well up in his subject, clear, and with a terseness which, not too brief, loses nothing of its grace.

